# Valproic acid potentiates curcumin-mediated neuroprotection in lipopolysaccharide induced rats

**DOI:** 10.3389/fncel.2014.00337

**Published:** 2014-10-21

**Authors:** Amira Zaky, Mariam Mahmoud, Doaa Awad, Bassma M. El Sabaa, Kamal M. Kandeel, Ahmad R. Bassiouny

**Affiliations:** ^1^Department of Biochemistry, Faculty of Science, Alexandria UniversityAlexandria, Egypt; ^2^Faculty of Medicine, Alexandria UniversityAlexandria, Egypt

**Keywords:** neuroinflammation, lipopolysaccharide (LPS), curcumin (Cur), valproic acid (VPA), let-7 miRNA

## Abstract

The etiology of neuroinflammation is complex and comprises multifactorial, involving both genetic and environmental factors during which diverse genetic and epigenetic modulations are implicated. Curcumin (Cur) and valproic acid (VPA), histone deacetylase 1 inhibitor, have neuroprotective effects. The present study was designed with an aim to investigate the ability of co-treatment of both compounds (Cur or VPA, 200 mg/kg) for 4 weeks to augment neuroprotection and enhance brain recovery from intra-peritoneal injection of (250 μg/kg) lipopolysaccharide-stimulated neuroinflammatory condition on rat brain cortex. Cortex activation and the effects of combined treatment and production of proinflammatory mediators, cyclooxygenase-2 (COX-2), APE1, and nitric oxide/inducible nitric oxide synthase (iNOS) were investigated. Neuroinflammation development was assessed by histological analyses and by investigating associated indices [β-secretase (BACE1), amyloid protein precursor (APP), presenilin (PSEN-1), and PSEN-2)]. Furthermore we measured the expression profile of lethal-7 (let-7) miRNAs members a, b, c, e, and f in all groups, a highly abundant regulator of gene expression in the CNS. Protein and mRNA levels of neuroinflammation markers COX-2, BACE1, APP, and iNOS were also attenuated by combined therapy. On the other hand, assessment of the indicated five let-7 members, showed distinct expression profile pattern in the different groups. Let-7 a, b, and c disappeared in the induced group, an effect that was partially suppressed by co-addition of either Cur or VPA. These data suggest that the combined treatment induced significantly the expression of the five members when compared to rats treated with Cur or VPA only as well as to self-recovery group, which indicates a possible benefit from the synergistic effect of Cur-VPA combination as therapeutic agents for neuroinflammation and its associated disorders. The mechanism elucidated here highlights the particular drug-induced expression profile of let-7 family as new targets for future pharmacological development.

## INTRODUCTION

Lipopolysaccharide (LPS) is the most abundant component within the cell wall of Gram-negative bacteria that has been extensively used in models studying inflammation. LPS initiates cellular receptor transduction through Toll-like receptor 4 (TLR4), binding of LPS to TLR4 elicits a signaling network including the activation of NF-κB through multiple mediators (Bode et al., 2012). NF-κB plays a crucial role in regulating the transcription of genes related to innate immunity and inflammation responses and several studies indicate its activation is controlled by reactive oxygen species (ROS) in immune modulation in monocytes (Asehnoune et al., 2004 and Park et al., 2004). Oxidative stress is associated with virtually all central nervous system pathogenesis, infectious, inflammatory, or degenerative in nature. Since brain homeostasis largely depends on integrity of blood–brain barrier (BBB) and oxidative stress associates BBB permeability alteration, brain is endowed in numerous antioxidant effectors via orchestrated actions of immune cells, vascular cells, and neurons that constitute neuroinflammation, that control and prevent the detrimental formation of ROS generated via different metabolic reactions (Dasuri et al., 2013; Xanthos and Sandkühler, 2014). Curcumin (Cur) has been shown to exhibit activity against various neurologic diseases; it is a potent inhibitor of reactive astrocyte expression and thus, prevents hippocampal cell death induced by kainic acid (Shin et al., 2007). Recent studies indicate that low doses of Cur is effectively disaggregate beta amyloid protein as well as prevents fibril and oligomer formation and hence has protective effect in treating Alzheimer’s disease (AD; Kulkami et al., 2009). Recent experimental researchers have shown protective effect of Cur in animal models of seizures (Du et al., 2009), and experimental model of epileptogenesis (Jyoti et al., 2009). Recently, Cur possess antidepressant activity and can modulate the release of serotonin and dopamine. Cur enhances the level of neurotrophic factors such as brain derived neurotrophic factor (BDNF; Wang et al., 2008)

Apurinic/apyrimidinic endonuclease (APE1) is essential for cell survival in mammalian cells, APE1 is also essential in early embryonic development (Meira et al., 2001; Izumi et al., 2005). APE1 is the second repair enzyme in base excision repair (BER)-pathway and hydrolyses the phosphodiester backbone immediately 5′ to an AP site to produce 3′ OH group and 5′deoxyribose-5-phosphate (Levin and Demple, 1990). APE1 knockdown in neurons accumulates significant oxidative DNA damage without efficient repair, which demonstrates that APE1 is essential for repair of oxidative DNA damage in neurons (Yang et al., 2010). In neurons, BER is the predominant mechanism for repair of oxidative DNA lesions. The major pathological features of AD are extracellular amyloid beta (Aβ) plaques and intracellular neurofibrillary tangles (NFTs). Moreover it has been reported that Aβ level differentially modulates APE1, a key enzyme in BER pathway, expression which may contribute to selective neuronal vulnerability in AD (Mantha et al., 2012). We have previously reported that APE1 expression is significantly reduced during neuroinflammation progression and restored by resveratrol treatment (Zaky et al., 2013).

Histone deacetylase inhibitors (HDAC inhibitors) promote the acetylation of histones, which are generally associated with transcriptional activation. HDACs inhibitors also increase the acetylation status and modulate the activity of a wide range of histone as well as non-histone proteins. HDAC inhibitors have been shown to confer neuroprotection in experimental models of various neurodegenerative diseases (Camelo et al., 2005; Gardian et al., 2005; Petri et al., 2006) even though the exact mechanisms underlying their neuro-protective actions are still elusive.

Moreover, valproic acid (VPA; 2-propylpentanoic acid sodium salt), a drug commonly used to treat seizures, has been shown to exert neuro-protective effect at therapeutic levels in cellular and animal models. In cultured neurons, VPA protects from glutamate-induced excitotoxicity, thapsigargin-induced endoplasmic reticulum stress, and LPS-induced dopaminergic neuronal death (Phiel et al., 2001; Kanai et al., 2004). Recently, VPA was shown to inhibit LPS-induced, microglia-mediated inflammation in midbrain neuron-glia cultures as described by inhibition of TNF-α secretion and NO production (Peng et al., 2005).

Alzheimer’s disease pathology is characterized by an accumulation of extracellular amyloid plaques composed of Aβ peptide fragment and intracellular NFTs composed of hyperphosphorylated protein tau, as well as neuronal loss in the hippocampus, temporal, and frontal lobes, increased inflammation, and oxidative stress (Serrano-Pozo et al., 2011)

MirSVR is a new machine learning method for ranking microRNA (miRNA) target sites by a down-regulation score. In a large-scale evaluation, miRanda-mirSVR is competitive with other target prediction methods in identifying target genes and predicting the extent of their down-regulation at the mRNA or protein levels. Importantly, the method identifies a significant number of experimentally determined non-canonical and non-conserved sites (Betel et al., 2010).

Altered biogenesis and/or function of miRNA are implicated in the various pathological processes including inflammation and neurodegeneration. In Alzheimer’s, miRNA profiling experiments have identified disease-specific miRNAs. Moreover a number of studies have linked differential miRNAs expression to pathology in AD such as deposition of amyloid plaques and NFTs, as well as more specific pathway interactions and regulatory functions of the amyloid pathway, including regulation of amyloid protein precursor (APP) and beta-site APP cleaving enzyme 1 (β-secretase, BACE1; reviewed in Gustaw-Rothenberg et al., 2010; Geekiyanage et al., 2012).

The aim of our study is to investigate the protective/therapeutic potential of Cur alone and in combination with histone deacetylase 1 (HDAC1) inhibitor, VPA, in LPS-induced rats. Also we are interested in studying the expression profiles of lethal-7 (let-7) miRNAs family members as signaling molecules in regulation of inflammatory enzymes cyclooxygenase-2 (COX-2) and inducible nitric oxide synthase (iNOS).

## MATERIALS AND METHODS

### ANIMALS AND ESTABLISHMENT OF NEUROINFLAMMATION EXPERIMENTAL MODEL

Fifty five male adult Sprague–Dawley (80–150 g) rats were used for the present study. The animals were supplied and maintained at medical research institute in which the European principles of laboratory animal ethics care were followed in all experimental protocols. Rats were maintained under controlled temperature (25 ± 2°C) and constant photoperiodic conditions (12:12-h daylight/darkness). The dams had free access to water and standard commercial chow containing 20% protein, 54% carbohydrate, 4.5% fiber, 4% lipids, 7% ash, and 10% moisture.

**FIGURE 1:**
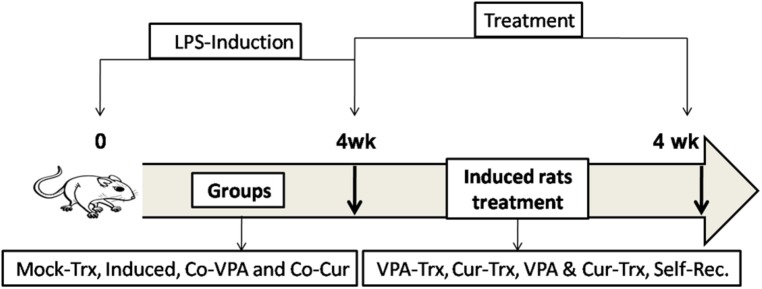
**Experimental design and groups classifications**.

Neuroinflammation induction was established by LPS injection. Experimental design and rats classifications as indicated in **Figure 1**, included the following groups: (1) mock-treated rats (mock-Trx) that received empty vehicle, (2) LPS-induced rats that received intra-peritoneal (IP) injection of 250 μg/kg LPS five times per week for 4 weeks, (3) Co Cur rats that received oral administration of 200 mg/kg Cur during LPS induction, (4) Co-VPA that were orally administered 200 mg/kg VPA in parallel to LPS. Treatment protocol involved oral administration of 200 mg/kg of Cur (Trx-Cur), VPA (Trx-VPA), or their combination (Trx-Cur + VPA) four times per week for 4 weeks. The Cur-VPA treated rats were administered the two doses at the same time. Moreover a group of LPS-induced rats were left untreated for the duration of 4-weeks in parallel to treated ones and referred as self-recovery to promote self-healing mechanism.

#### Samples collection

Blood was collected from each group at time intervals of 2 and 4 weeks of LPS administration and after treatment by retro-orbital bleeding or during sacrifice. From each group 4–6 rats were killed by cervical dislocation and sera were collected and stored at -80°C for further analyses. Brain tissues were removed, washed with cold 0.9% NaCl and dissected into different lobes; mid brains were stored in RNA later for extraction of total RNA and the remaining section was rapidly frozen with liquid nitrogen and stored at -80°C for preparation of total and nuclear extracts (NEs), and enzyme assays. A whole brain was removed for histochemical studies by fixation with 10% buffered-saline formalin.

#### Histopathological analysis

Formalin fixed brains were processed and cortex tissues were stained with hematoxylin and eosin (H&E) and examined for any histopathological changes. Pathological diagnosis of each brain specimen was assessed and analyzed by specialized histopathologist.

### DETERMINATION OF OXIDATIVE STRESS MARKERS

#### Sera total antioxidant capacity

Blood was collected without using an anticoagulant, allowed to clot for 30 min at 25°C then centrifuged at 3000 rpm for 15 min at 4°C. Serum was collected and stored at -80°C if not assayed on the same day. The assay was preformed according to method described before (Koracevic et al., 2001).

#### Reduced glutathione (GSH) level

Dissected parts of the brain cortex tissues (10% w/v) were washed with saline solution minced and homogenized in ice cooled homogenization buffer (1.15% KCl, 0.01 M sodium phosphate buffer pH 7.4). The homogenates were centrifuged at 10,000 rpm for 20 min at 4°C and the supernatants were used for determination of glutathione (GSH) and protein contents. GSH contents were determined as described by Ellman (1959). Briefly, Ellman’s reagent [5,5′, dithiobis (2-nitrobenzoic acid), DTNB ] reacts with GSH to give 2-nitro-5-thiobenzoic acid, a yellow colored product with a maximum absorbance at 412 nm, GSH level is expressed as mg/g. tissue.

#### Lipid peroxidation

Frozen sections of brain tissues (10% w/v) were washed with saline solution, minced, and homogenized in ice cooled buffer (50 mM potassium phosphate pH 7.5), the homogenates were centrifuged at 10,000 rpm for 20 min at 4°C and the supernatants were used for the assay. Levels of lipid peroxidation were determined according to Ohkawa et al. (1979) colorimetric method. Thiobarbituric acid (TAB) reacts with malondialdehyde (MDA) in acidic medium at 95°C for 30 min to form TAB reactive product to give pink color that is measured at 534 nm and MDA content is expressed in nmol/g tissue.

#### Superoxide dismutase activity (SOD)

Brain tissue was homogenized in ice cooled buffer (100 mM potassium phosphate, pH 7.0 containing 2 mM EDTA) per gram tissue. Superoxide dismutase activity (SOD) was assayed according to Nishikimi et al. (1972). The assay depends on the ability of the enzyme to inhibit the phenazine methosulphate-mediated reaction of nitroblue tetrazolium dye. Phenazine methosulphate (0.1 mM/L) was diluted 500 times immediately before use in distilled water. In addition to working reagent was freshly prepared by mixing phosphate buffer (50 mM/L) pH 8.5, nitroblue tetrazolium (1 mM/L), and NaOH (1 mM/L) in ratio of 10 + 1 + 1. Reaction mixture prepared by mixing the working reagent and samples, then initiated by addition of diluted phenazine methosulphate according to the protocol indicated ratios.

### TOTAL RNA ISOLATION AND REVERSE TRANSCRIPTASE POLYMERASE REACTION (RT-PCR)

Total RNA was extracted from frozen brain tissues according to Chomczynski and Sacchi (1987) using GStractTM RNA Isolation Kit II Guanidinium Thiocyanate Method (Maxim Biotech Inc., USA). Quality of RNA preparations were confirmed by calculating 260/280 ratio for detection of protein contamination and by running samples on agarose to confirm that the samples are DNA-free. Alterations in the target mRNA levels of genes relevant to microglia activation and neuroinflammation were determined using either semi-quantitative reverse-transcriptase PCR (semi-qRT-PCR) or quantitative real time RT-PCR (qRT-PCR).

#### Semi-quantitative RT-PCR

Semi-quantitative RT-PCR was performed using one-step RT-PCR (RT/PCR Master Mix Gold Beads, BIORON). The cDNA was synthesized and used for amplification of target gene(s). Briefly, total RNA (1–3 μg) and random primer (3 μM) mixture were denatured at 70°C for 5 min and placed on ice. The incubated mixture was added to the RT/PCR Gold mix that contains all the components necessary for cDNA synthesis and amplification in one tube. The cDNA synthesis reaction was performed at 42°C for 60 min then 5 min at 94°C for RTase inactivation. The primers then subjected to PCR cycles, each cycle consisting of denaturation, annealing, and extension. Annealing temperature and time was optimized for each primer/template combination. We investigated the expression of neuroinflammatory markers APP, BACE1, γ-secretase (presenilin; PSEN-1 and PSEN-2) and iNOS expressions using the following primers sets: APP; F-AGAGGTCTACCCTGAACTGC- R- ATCGCTTACAAACTCACCAAC (154 bp), BACE1; F-CGGGAGTGGTATTATGAAGTG- R-AGGATGGTGATGCGGAAG (320 bp), PSEN-1; F- GGATGGGCAGCTAATCTATAC- R- CCTTCAGCCATATTCACCAAC (576), PSEN-2; F-GAG CAG AGC CAA ATC AAA GG- R-GGGAGAAAGAACAGCTCGTG (188 bp), iNOS; F-GTGTTCCACCAGGAGATGTTG- R-CTCCTGCCCACTGAGTTCGTC (576 bp) and for validation we used β-actin: F-GGC ATC CTG ACC CTG AAG TA- R-GCCGATAGTGATGACCTGACC (565 bp). Products of RT-PCR were separated on agarose gel, visualized and documented using ChemiDoc-It^®;^2 Imager then analyzed with VisionWorksLS Acquisition and Analysis Software for determinations of relative bands intensity.

#### Quantitative RT-PCR assay

Quantitative real time RT-PCR was used to measure the mRNA levels of APE1, let-7a, b, and c. analyses were performed using miScript II RT Kit (Qiagen) according to the manufacture guidelines. The primers for APE1 were F-GCTTGGATTGGGTAAAGGA, R-TTCTTTGTCTGATGGAGCTG, COX-2; F- AGGCCTCCATTG ACCAGA- R- TCATGG TAGAGGGCTTTCAAC, β-actin; F-CCGACAGGATGCAGAAGG-3′ and R-GGAGTACTTGCGCTCAGGAG, let-7a; TGA GGT AGT AGG TTG TAT AGTT, let-7b; TGA GGT AGT AGG TTG TGT GTTT, let-7c; TGAGGTAGTAGGTTGTATGGTT and U6; F-GGAACGATACAGAGAAGATTAGC, R- AAATATGGAACGCTTCACGA. Gene expression results of indicated genes and miRNAs were normalized to β-actin and U6, respectively, fold difference calculated as described before (Livak and Schmittgen, 2001).

### PREPARATION OF CYTOSOLIC AND NUCLEAR EXTRACTS AND WESTERN BLOTTING

The extraction of cytosolic and nuclear rich fractions was performed according to Dignam et al. (1983) procedure. Briefly brain tissues were homogenized using hypotonic buffer (10 mM HEPES buffer, pH 7.5 containing 10 mM KCl, 3 mM NaCl, 3 mM MgCl2, 1 mM EDTA, 1 mM EGTA, 2 mM DTT, 2 mM PMSF, and protease inhibitor cocktail) and kept on ice for 15 min. The supernatants (cytoplasmic extracts) were collected by centrifugation then stored at -80°C and the pellets were washed in 200 μl of hypotonic buffer and re-centrifuged. The pellets nuclei were re-suspended in 100 μl of ice-cold NE buffer (20 mM HEPES buffer, pH 7.5 containing 25% glycerol, 500 mM KCl, 1 mM MgCl2, 1% NP-40, 1 mM EDTA, 2 mM DTT, 2 mM PMSF, and protease inhibitor cocktail), and incubated on ice for 20 min, with occasional mixing, then centrifuged at 14,000 × *g* for 15 min at 4°C. The resulting supernatants, nuclear extracts, were collected and stored at -80°C for further analysis. Primary antibody to APE1 (sc-17774) and COX-2 (sc-7951) were used and equal loading was confirmed by probing with β-Actin (sc-81178) monoclonal antibody.

### STATISTICAL ANALYSIS

Experiments were repeated two or more times independently and graphs are represented as mean ± SD. The difference between groups was analyzed by one-way analysis of variance (ANOVA) and the difference considered significant either at *p* < 0.01 or at *p* < 0.001 when compared to mock-treated group.

## RESULTS

### HISTOLOGICAL ANALYSES

**FIGURE 2:**
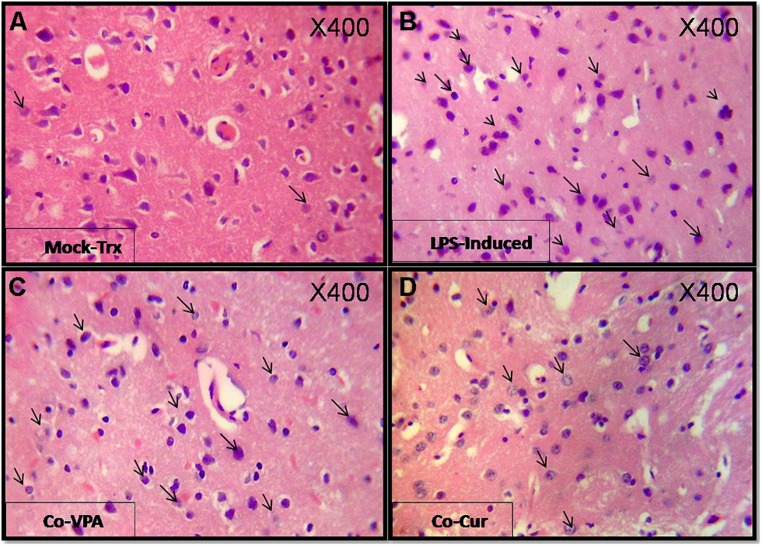
**Shows H&E (400X) staining of Cortex sections from different experimental groups: **(A)** shows photographs of mock-treated with normal parenchyma and architecture. (B)** shows lipopolysaccharide (LPS)-induced cortex sections featuring an aggregate of mature lymphocytes an effect that was partially inhibited by co-administration of Valproic acid (VPA; **C**) and Curcumin (Cur; **D**). Apparently Co-Cur group showed sparse lymphocytic infiltrate compared to mock-treated. Arrows indicate the activated lymphocytes.

**FIGURE 3:**
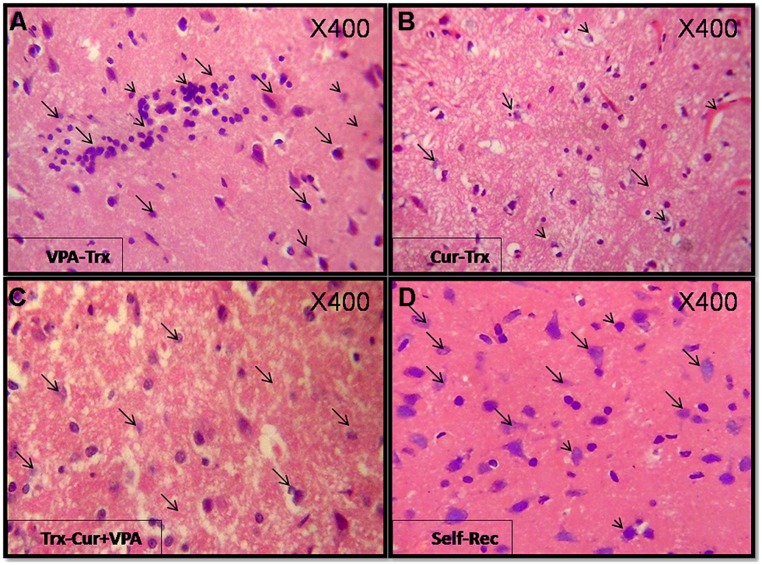
**Show H&E (400X) stained sections for the treated groups: Cur, VPA, and VPA-Cur as well as the self-recovery group that received no treatment for 1 month.** The represented photographs indicate that VPA acid treated rats **(A)** featured moderately intense lymphocytic infiltrate and Cur treatment **(B)** induced suppression of the inflammatory response as depicted by negligible lymphocytic infiltrate as marked by the arrows. Moreover VPA-Cur combination **(C)** exerted the most anti-inflammatory effect by showing no inflammatory infiltrate compared to self-recovery group **(D)** that contain sparse lymphocytic infiltrate as indicated by the arrows.

Brain tissues of mock-treated, LPS-induced, Co-treated, and treated groups were examined using H&E staining as shown in (**Figure 2**) for confirmation of progression to neuroinflammation. The histopathology of cortex tissue from rats induced with LPS and co-treated with VPA and Cur showed some protection effect against neuroinflammation (**Figures 2C,D**). Although treatment with VPA exerted inhibitory effect on neuroinflammation but Cur showed more potent effect than VPA (**Figures 3A,B**). Interestingly combination of both Cur and VPA induced strong synergistic effect showing no significant neuroinflammation (**Figure 3C**) compared to self-recovery group (**Figure 3D**), which is consistent with the biochemical markers profile.

### CURCUMIN COMBINATION WITH VALPROIC ACID AMELIORATES LPS-INDUCED OXIDATIVE STRESS

**FIGURE 4:**
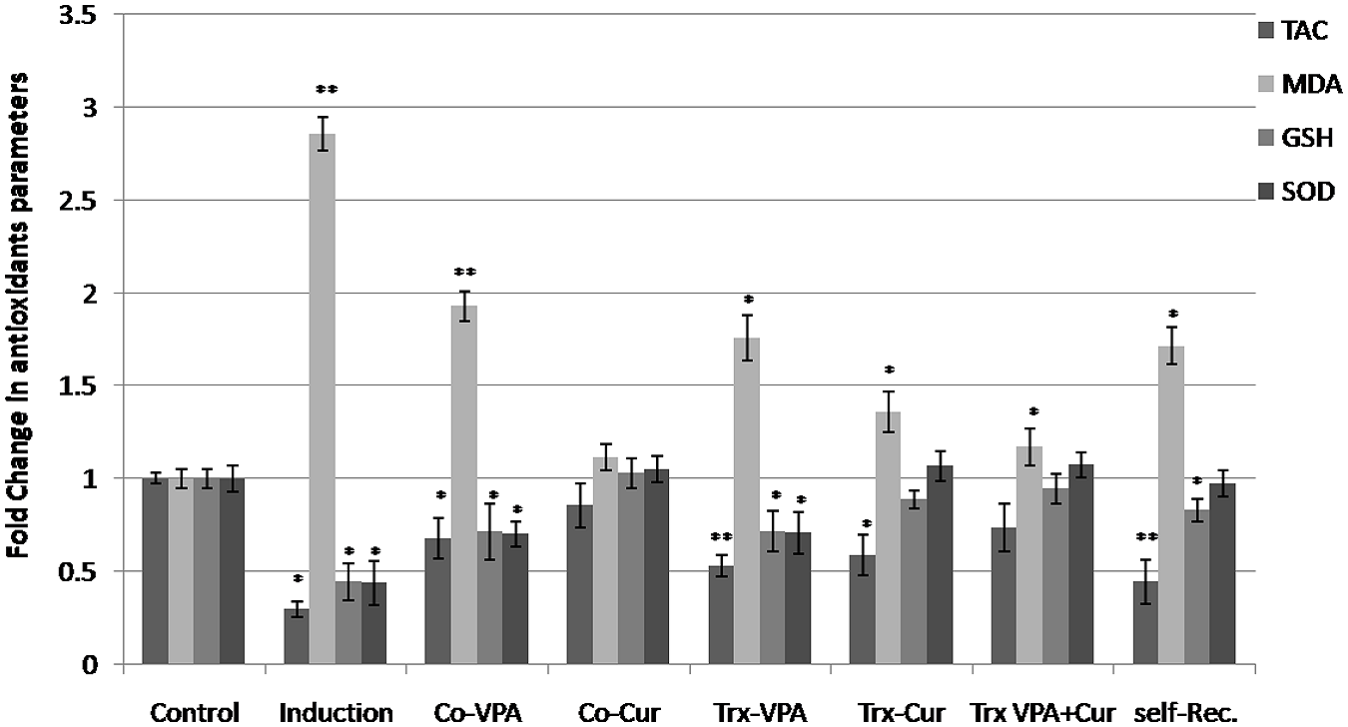
**Fold alterations in antioxidant capacity versus mock-treated rats.** Oxidative stress related markers total antioxidant capacity (TAC), glutathione (GSH), and malondialdehyde (MDA) levels as well as superoxide dismutase activity (SOD) activity were measured in the different experimental groups. LPS-induced an overall reduction in antioxidant defense system as indicated by significant (*p < 0.01) decrees in TAC, GSH, and SOD and significant (**p < 0.01) elevation in MDA content versus mock-treated rats (mock-Trx). Co-administration of VPA did not improve oxidative stress markers. On the contrary, Cur co-administration inhibited LPS-induced neurotoxicity markedly by maintaining all parameters near normal values. Moreover treating induced rats with cur but not with VPA restored GSH content and SOD activity. VPA addition to Cur exerted the most antioxidant effect on neuroinflamed rats. An improvement in GSH level and SOD activity was also observed in Self-Rec rats.

Oxidative stress is a hallmark of inflammatory mechanism. Herein we measured oxidative stress parameters represented by sera total antioxidant capacity (TAC), GSH, and MDA levels as well as SOD activity in the different experimental groups. Results are presented as fold change from the mock-treated group. An overall significant decline (*p* < 0.01) in the sera TAC, GSH level and SOD activity associated with significant elevation in MDA level (*p* < 0.001) were observed in LPS-induced brains (**Figure 4**). In parallel to histological observations, co-administration of VPA or Cur ameliorated LPS-induced neurotoxicity with most prominent effect upon Cur co-administration. Furthermore Cur treatment markedly improved both GHS level and SOD activity (**Figure 4**). Obviously VPA treatment was not effective in inhibiting LPS-induced oxidative stress as indicated by significant elevation in MDA (*p* < 0.01) accompanied with significant reduction in TAC, GSH level, and SOD activity (*p* < 0.001 and *p* < 0.01, respectively; **Figure 4**). Interestingly, adding VPA to Cur potentiated Cur effect and restored the oxidative stress markers back to normal when compared to mock-Trx. Restoration of GSH level and SOD activity in the self-recovery group indicated that shifting the oxidative stress balance is essential during physiological wound healing process (**Figure 4**).

### EXPRESSION PROFILE OF NEURO-INFLAMMATION MARKERS

**FIGURE 5:**
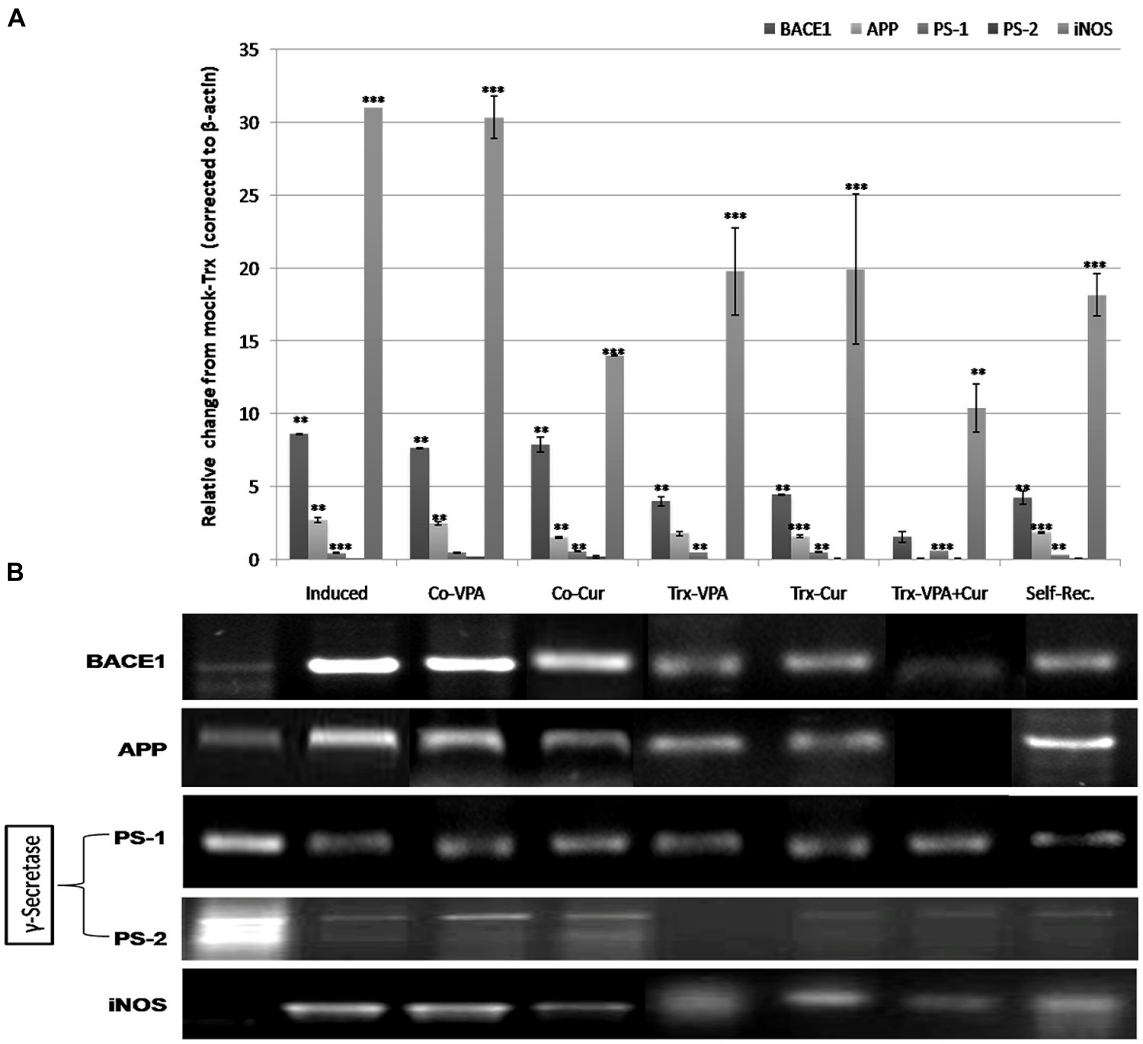
**Gene expression profiles of neuro-inflammation modulated genes assessed by reverse transcriptase (RT-PCR) analysis.** Total mRNA isolated from rats’ brain tissues were analyzed by semi-qRT-PCR for (β-secretase (BACE1), amyloid protein precursor (APP), PS-1, PS-2, and inducible nitric oxide synthase (iNOS) levels on agarose gel **(B)**. Quantifications of the bands on the gel using VisionWorksLS Acquisition and Analysis Software indicated that LPS induced significantly (**p < 0.01) the expression of BACE1, APP, and iNOS accompanied with reduction in PS-1 and PS-2 (***p < 0.001) genes compared to mock-Trx **(A)**. Co-VPA (200 mg/kg) treated rats showed no marked decrease in BACE1 and APP levels compared to LPS-induced rats, but still significantly higher (*p < 0.01) than control. Co-Cur administration (200 mg/kg), VPA and Cur treatments induced reduction in BACE1, APP, and iNOS expression level compared to LPS-induced rats **(A)**. A remarkable decrease in BACE1, APP, and iNOS level in VPA-Cur treated rats compared to LPS-induced group (but still significant from control at *p* < 0.01 and *p* < 0.001 respectively; **A**). Self-recovery rats showed no improvement in APP, BACE1, or iNOS levels.

The relevance of neuroinflammation to Alzheimer’s pathology has been elucidated by different reports. Microglial activation has been shown in regions associated with Aβ deposition. In the current study we examined the expression of BACE1, APP, and γ-secretase subunits (PSEN-1 and PSEN-2) as the major factors implicated in Aβ deposition as well as the inflammatory enzymes iNOS, COX-2, and APE1. Total RNA from cortex tissues were extracted and mRNAs were analyzed by semi-qRT-PCR for the indicated genes. LPS-induced significantly BACE1 and APP along with the inflammatory enzyme iNOS expressions (around 8-, 2-, and 30-folds, respectively) when calculated in reference to the expression level in mock-Trx (**Figure 3C**). In addition, LPS reduced PSEN-1 and -2 expressions significantly (*p* < 0.001). Co-addition of either VPA or Cur ameliorated LPS effect by around 10 and 30% respectively (**Figure 5B**). Moreover treatment of induced rats with Cur is more effective than VPA in reducing BACE1, APP, and iNOS expressions (*p* < 0.01) and increasing PSEN-1 and -2 levels (**Figures 5A,B**). We also show that adding VPA potentiates Cur-mediated anti-inflammatory properties and significantly reduced BACE1, APP (*p* < 0.001), and iNOS (*p* < 0.01) when compared to control and self-rec. groups (**Figure 5B**).

**FIGURE 6:**
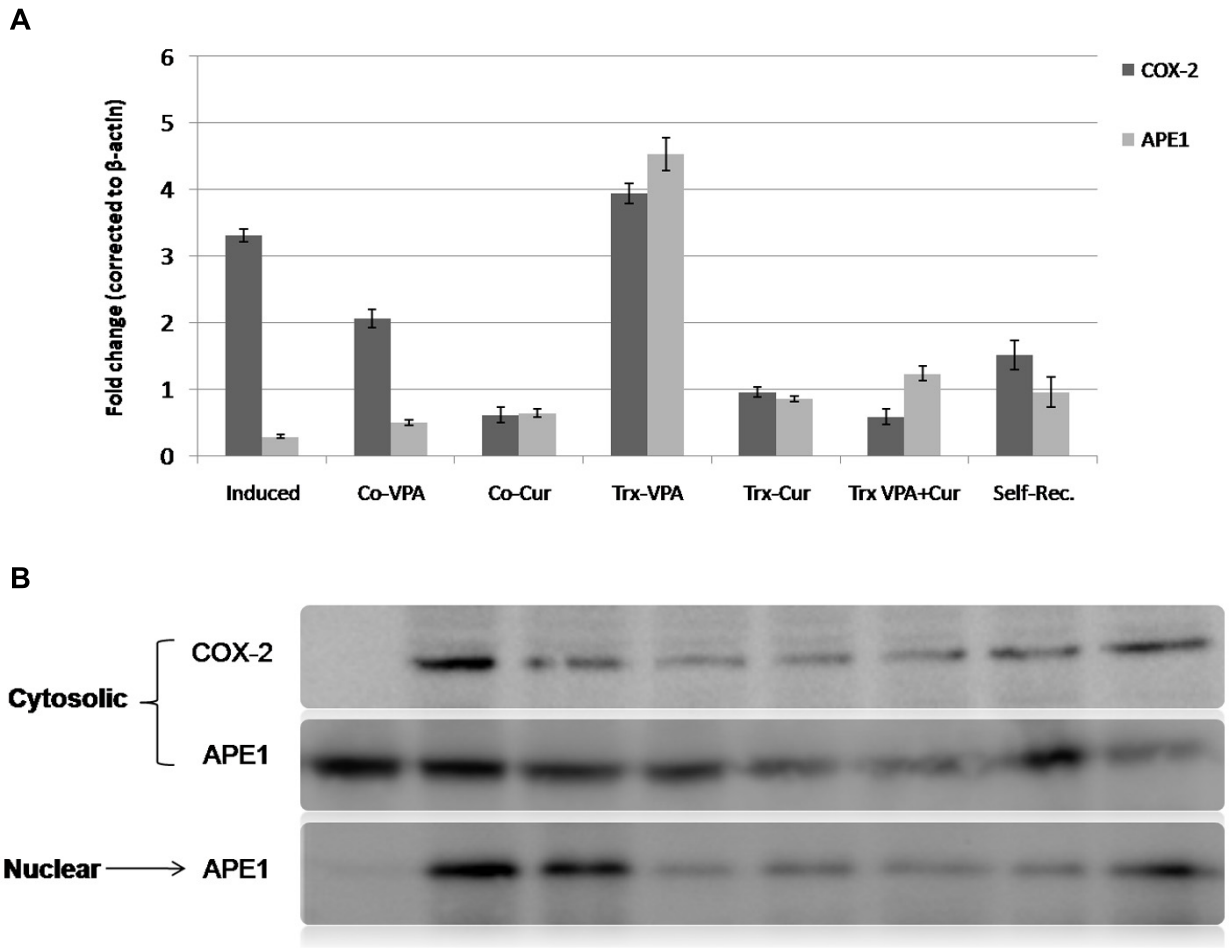
**Quantitative real time RT-PCR (qRT-PCR) and western blotting analysis of APE1 and COX-2 expressions.** mRNA and total proteins extracts were prepared (as described in the methods section) from mock-treated and LPS-induced as well as co-treated and treated groups. **(A)** and **(B)** showed a significant elevation (**p < 0.001) in cyclooxygenase-2 (COX-2) level of LPS-induced group as well as VPA-treated and co-VPA treated groups in comparison with mock-treated group versus reduction in COX-2 level in self-recovery group. A remarkable reduction (*p < 0.01) in COX-2 level in Co-Cur treated, Cur-Treated and VPA-Cur treated groups versus LPS-induced group. A remarkable reduction in APE1 in induced versus mock-treated group. Data revealed an elevation of APE1 in treated and Co-treated groups as well as self-recovery group but especially in VPA-treated group versus LPS-induced rats.

We have previously reported the involvement of APE1 down-regulation during the pathogenesis of neuroinflammation and maintaining high APE1 expression is associated with neuro-protection (Zaky et al., 2013). Cur has been reported to regulate COX-2 both at transcriptional and protein levels. In the current study we investigated APE1 and COX-2 as key inflammatory enzymes by real time-PCR (qRT-PCR) and western blotting. LPS induction for 4 weeks increased COX-2 expression by up to threefold and reduced APE1 by around 70% when compared to control (**Figure 6A**). Continuous administration of Cur with LPS inhibited significantly the brain toxicity by reducing COX-2 and maintaining high APE1 expressions. On the contrary, VPA co-administration did not improve COX-2 or APE1 levels. Although Cur treatment significantly inhibited COX-2 and induced APE1expressions (up to normalization; **Figure 6A**), but VPA-Cur combination was more effective on inhibiting COX-2 and inducing APE1. In consistent with COX-2 gene expression results (qRT-PCR), western blotting analysis showed similar pattern of cytosolic COX-2 protein (**Figure 6B**).

Because LPS-induced neurotoxicity is associated with oxidative stress and DNA mutation that activate APE1 nuclear translocation, therefore we investigated nuclear versus cytosolic distribution of APE1 protein. The results showed high nuclear localization in LPS-induced and Co-VPA administered rats. In parallel to Cur effects on antioxidant capacity and neuroinflammation markers, we observed marked reduction in nuclear APE1 level upon continuous supply of Cur during induction (**Figure 6B**). Likewise Cur only and in combination with VPA was shown to be effective in switching APE1 localization to cytoplasm versus VPA-treated and self-Rec groups (**Figure 6B**).

### MODULATION OF FIVE MEMBERS OF let-7 FAMILY miRNAs

**FIGURE 7:**
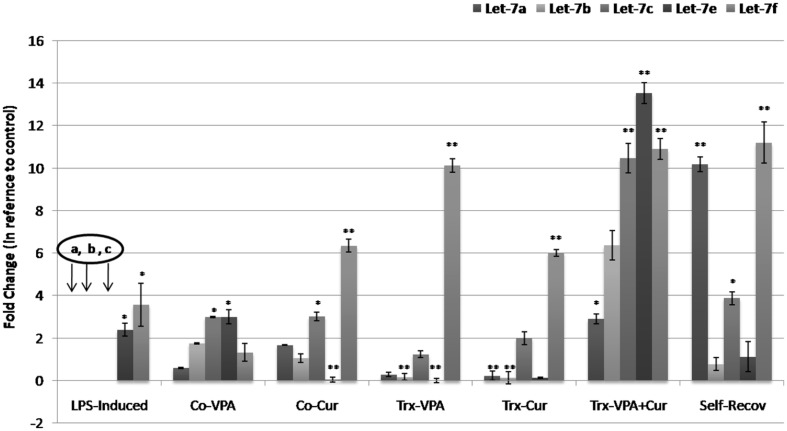
**Quantitative real time RT-PCR analyses of let-7 miRNAs family expression profile in different experimental groups.** In our study we measured the alterations in the expression levels of five different types of let-7 miRNAs (Let-7 a, b, c, e, and f). Mock-treated, LPS-induced, co-treated and treated rat’s miRNAs extracts were subjected to quantitative real time polymerase chain reaction (qRT-PCR) of target genes expression. Completed reductions (under detection limit) in let-7a, b, and c levels were observed in LPS-induced rats, the effect that was ameliorated by co-administration of VPA or Cur (**p < 0.001). Moreover VPA+Cur combination for treating induced rats significantly (*p < 0.01) induced the five members of let-7 family in comparison to LPS-induced rats.

microRNAs are considered crucial regulators of cellular immunity and functions. Accumulating data from different studies have highlighted diverse alterations in miRNAs biogenesis and regulatory role in inflammatory diseases including neurodegeneration. miRNA let-7 family members are highly expressed in central nervous system and were shown to play crucial role in cell development and differentiation. Therefore we analyzed the expression profile of five members of let-7 family (a, b, c, e, and f) in the different experimental groups. The results show that let-7 a, b, and c were under detection level in LPS induced rats, an effect that was countered by either co-VPA or co-Cur incorporation (**Figure 7**). We also observed unique pattern of drug-induced differential expression of the five miRNAs types. Moreover Cur or VPA treatment of induced rats exhibited the same pattern of inducing significantly let-7c and f compared to Cur-VPA treated rats that showed significant restoration in the expression of the five members (**Figure 7**). Evidently we observed significant elevations in let-7 a, c, and f in self-rec. group which confirms the implication of these let-7 members in self-healing mechanism.

## DISCUSSION

In the present study, we observed the alteration of the inflammatory response in male adult Sprague–Dawley rats brain cortex and investigated the protective versus therapeutic effects of Cur alone or in combination with HDAC1 inhibitor, VPA on attenuating LPS-induced neuroinflammation in rats. We focused on Cur, which is used as a food additive, due to its diverse pharmacological activities, very low toxicity and widespread availability. The therapeutic potential of VPA in central nervous system diseases is also gaining support. We administered Cur and/or VPA (200 mg/kg) for 4 weeks and observed production of proinflammatory mediators, COX-2, APE1, and nitric oxide/iNOS. The combined therapy decreased the production and mRNA expression of the inflammatory TNF-α and interleukin (IL-6; data not shown). In addition, expression of iNOS and COX-2 were decreased by cotreatment with Cur and VPA. In conclusion, these results show that the anti-inflammatory properties of Cur and VPA potentially result from the inhibition of COX-2, iNOS, IL-6, TNF-α, and NO through activation of NF-κB. These results impact the development of potential health products for preventing and treating inflammatory diseases.

Consistent with the inhibition of pro-inflammatory mediators by Cur and VPA, we observed an anti-inflammatory role of Cur via inhibition of NF-κB pro-inflammatory pathways. Therefore, the NF-κB pathway is potentially involved in the anti-inflammatory therapeutic effects of Cur in a variety of neuropathologies. In support of this, Cur has been found to block the LPS-mediated induction of COX-2 through inhibition of NF-κB and STAT3 (Kim et al., 2003; Kang et al., 2004). Although the beneficial effects of combined therapy can be observed and conclusive under various experimental conditions, the effects of Cur and VPA in rat cortex cells exposed to LPS remain to be fully elucidated. Further experiments are required to explore the detailed mechanisms underlying this process. Regardless of the mechanism, the data presented in this study may assist future studies that aim to determine the therapeutic potential of Cur and VPA.

In a recent study, neuroinflammation was induced by bilateral intracerebroventricular (ICV) administration of LPS (Tyagi et al., 2008). They studied the proinflammatory cytokines (TNF-α and IL-1β), acetylcholinesterase (AChE) activity, MDA, and reduced GSH as markers for neuroinflammation, cholinergic activity, and oxidative stress, respectively, in different brain regions at different time points after LPS injection. They found enhanced AChE activity with these inflammatory markers after LPS administration indicates a possible relationship between neuroinflammation and cholinergic system during the development of neurodegenerative diseases. It was found also that cholinergic agonists inhibit cytokine synthesis and protect against cytokine-mediated diseases (Tracey, 2007).

Findings from an experiment of selective serotonin reuptake inhibitors (SSRIs) by Waiskopf et al. (2014) suggested a possible interaction with both TLR4 responses and cholinergic signaling through as yet unclear molecular mechanism(s). They also demonstrated reduced LPS-induced pro-inflammatory IL-6 and TNF-α in human peripheral blood mononuclear cells preincubated with antidepressant fluoxetine. Furthermore, they showed that fluoxetine intercepts the LPS-induced decreases in intracellular AChE-S and that AChE-S interacts with the NF-κB-activating intracellular receptor for activated C kinase 1 (RACK1). This interaction may prevent NF-κB activation by residual RACK1 and its interacting protein kinase PKCβII. These findings attribute the anti-inflammatory properties of SSRI to surface membrane interference with leukocyte TLR4 activation accompanied by intracellular limitation of pathogen-inducible changes in AChE-S, RACK1, and PKCβII (Waiskopf et al., 2014).

Also, Sailaja et al. (2012) reported that chromatin structure and histone modifications are causally involved in this transcriptional memory. Specifically, the AChE gene is known to undergo long-lasting transcriptional and alternative splicing changes after stress. Their findings provide further support and underscore the importance of better understanding for the notion that chromatin regulation is an important mechanism controlling long-term adaptive changes in the brain associated with complex psychiatric conditions.

In the present study we assessed the effect of the HDAC1 inhibitor VPA by measuring alterations at histological, biochemical, and molecular levels. The early stage of asymptomatic stage, reached by LPS with a modified dose (250 μg/kg five times per week for 4 weeks) was established to explore the mechanism of action of Cur, VPA, and VPA-Cur combination on the attenuation of LPS-induced neuroinflammation. Histological analyses indicated that co-administration of either VPA or Cur inhibited LPS-induced lymphocyte infiltrations in the cortex tissues. However, treatment of induced rats with VPA, Cur or their combination for 4 weeks post LPS-induction, exerted neuronal recovery, but the most significant improvement was observed upon treatment with VPA-Cur combination as confirmed by H&E staining.

Altered oxidative stress levels determined in our experiments verify the neuroinflammation responses as explained by Ferger et al. (2010). Ngkelo et al. (2012) indicated the implication of ROS in the mechanism of TLR4 activation by LPS. The biological effects of Cur are mainly derived from its ability to either bind directly to various proteins such as COX-2 or its ability to modulate the intracellular redox state (Hong et al., 2004). In parallel, we investigated the anti-oxidant effect of VPA, Cur, and VPA-Cur combination by measuring tissues MDA and GSH levels as well as SOD activities. Our results clearly demonstrate that co-Cur administration inhibited significantly LPS-induced brain toxicity and oxidative stress compared to co-VPA group. In addition to, treatment with Cur alone or in combination with VPA restored oxidative stress balance significantly (close to normalization) compared to both VPA-treated and self-recovery groups.

Several lines of investigation support the notion that the pathogenesis of AD is related to progressive accumulation of Aβ protein, as a result of an imbalance between the levels of Aβ production, aggregation and clearance. It has been shown that the abnormal processing of APP by β and γ-secretase protease enzymes is a key event in the development of Alzheimer’s disease (AD) neuropathology (Sastre et al., 2003), resulting in an increase in the generation of the 42 amino acid form of Aβ peptide which aggregates to form the insoluble amyloid plaques. Hauss-Wegrzyniak and Wenk (2002) demonstrated that LPS induced extracellular deposition of beta-amyloid fibrils into the hippocampus suggesting that there is a close connection between amyloidogenesis and LPS-induced neuro-inflammation and LPS-induced increase of APP level. The γ-secretase complex has not yet been fully characterized but minimally consists of four individual proteins including PSEN. Therefore we detected the expression profile of neuroinflammation-related markers; APP, BACE1, and γ-secretase subunits (PSEN-1, PSEN-2) using semi-qRT-PCR in cortical region of rat’s brain tissues. We observed marked induction of BACE1 and APP expressions in the cortex tissues of LPS-induced rats along with clear reduction in the expression levels of both γ-secretase subunits. Consistently, we observed marked suppression in BACE1 and APP expressions upon co-Cur administration, Cur-treatment and more significantly in VPA-Cur treated rats versus marked induction of PSEN-1 and PSEN-2 subunits levels in the same groups.

Several lines of investigation support the notion that the pathogenesis of AD is related to progressive accumulation of Aβ protein, as a result of an imbalance between the levels of Aβ production, aggregation, and clearance. It has been shown that the abnormal processing of APP by β and γ-secretase protease enzymes is a key event in the development of AD neuropathology (Sastre et al., 2003), resulting in an increase in the generation of the 42 amino acid form of Aβ peptide which aggregates to form the insoluble amyloid plaques. Hauss-Wegrzyniak and Wenk (2002) demonstrated that LPS induced extracellular deposition of beta-amyloid fibrils into the hippocampus suggesting that there is a close connection between amyloidogenesis and LPS-induced neuro-inflammation and LPS-induced increase of APP level. The γ-secretase complex has not yet been fully characterized but minimally consists of four individual proteins including PSEN. Therefore we detected the expression profile of neuroinflammation-related markers; APP, BACE1, and γ-secretase subunits (PSEN-1, PSEN-2) using semi-qRT-PCR in cortical region of rat’s brain tissues.

We observed marked induction of BACE1 and APP expressions in the cortex tissues of LPS-induced rats along with clear reduction in the expression levels of both γ–secretase subunits. Consistently, we observed marked suppression in BACE1 and APP expressions upon co-Cur administration, Cur-treatment and more significantly in VPA-Cur treated rats versus marked induction of PSEN-1 and PSEN-2 subunits levels in the same groups.

Consistently in the present study, by investigating APE1 expression in LPS-neuroinflammation established model, the results revealed significant reduction of both APE1 mRNA level, and intracellular protein distribution (cytosolic versus nuclear) compared to mock-treated rats. Furthermore, we show that Cur co-administration maintained significantly elevated APE1 level during the course of LPS-induction compared to induced rats with more nuclear localization. Furthermore we found that VPA-Cur treatment was more effective than either Cur or VPA treatments alone in restoring high APE1 expression profile and inducing more cytosolic localization. The intracellular localization of the multifunctional APE1 has been closely correlated to its various functions, hence nuclear versus cytosolic distribution level reflects a vital role in BER or in redox co-activation of different transcription factors. This suggests that Cur and VPA-Cur combination actions are mediated, in part, by maintaining elevated APE1 level and shifting the intracellular balance to more cytosolic localization, a state that mimic normal condition.

In agreement with Peng et al. (2005) that showed that VPA may cause MKP-1 activation to dephosphorylate p38MAPK and JNK, leading to decrease in p65 and C/EBPb binding to the COX-2 promoter region and COX-2 down-regulation in LPS-stimulated bEnd.3 cells, our data established that co-expression of inflammatory proteins COX-2, iNOS and amyloid peptides were higher in the LPS-treated rats. However, Cur/VPA decreased the LPS-induced expressions of COX-2, iNOS, and amyloid peptides. Cur administered to rats either alone or in combination with VPA exhibited the most significant inhibitory effect on COX-2 and iNOS expressions in their brain tissues extracts.

Recently miRNAs have been identified as crucial regulators of immune cell development and function. Deregulated miRNAs contribute to the development of various diseases, for example, cancer, cardiovascular, or neurological diseases (Bonauer et al., 2010; Gascon and Gao, 2012; Thum, 2012).

Neurodegeneration is characterized by neuronal loss of specific neuronal circuits associated with cognitive and motor functions and by changes in miRNA levels in the nervous tissue and in the periphery. Recent reports of microRNA modulators of both neuronal and immune processes (termed NeurimmiRs) predict therapeutic potential for manipulating NeurimmiR levels in diseases affecting both the immune system and higher brain functions, such as AD, Parkinson’s disease (PD), multiple sclerosis (MS), and anxiety-related disorders (Soreq and Wolf, 2011). Manipulating NeurimmiR control that function within both the nervous and the immune systems, over the immune contributions to cognitive pathways may offer new therapeutic targets. Amongst them let-7 family miRNA that were reported to be broadly expressed across all differentiated tissues and their expression is tightly controlled during embryonic stem cells differentiation. In our study, we screened in particular, five types of let-7 miRNAs family which are; let-7a, let-7b, let-7c, let-7e, and let-7f for possible modulation during the course of induction, protection, and treatment. Interestingly we observed an overall altered expression profile in the five types of let-7 miRNAs in induced versus protected and treated rats. However, let-7a, b, and c levels were undetectable when assessed using qRT-PCR in induced group, a significant expression was observed in Co-Cur and Co-VPA administered groups, which suggest their implication in neural protection. In addition, their levels were up-regulated up to 3, 6, and 11 folds consequently in VPA-Cur treated group which confirm their involvement in neural recovery of inflamed brain tissues.

A major question of our study was to delineate to what extent miRNA changes accompany disease and disease progression. miRNAs have recently been involved mostly in neurodegenerative disorders including AD (Lau et al., 2013). Their work reveal that most of the recorded expression changes in miRNAs are brain area specific, with 10 miRNAs deregulated in the hippocampus and prefrontal cortex in late onset AD. It is also likely that several miRNA changes recorded in our human brain samples are related to some neuroinflammatory changes occurring during disease, especially 132-3p which mediates anti-inflammatory signaling (Shaked et al., 2009). The targeting of acetylcholinesterase by miR-132-3p may be indeed relevant for the association of the enzyme activity with amyloid load in late onset AD (Alkalay et al., 2013; Lau et al., 2013).

Consistent with obtained results of the five members of let-7expression profile in the different experimental group, by applying mirSVR online data base for their mRNA targets prediction, we uncovered COX-2 gene (NM_011198) as a target for the five members while let-7b only regulates the expression of iNOS (NM_012611) gene. This explains the observation of let-7a, b, and c disappearance in induced group that was accompanied with threefold induction inCOX-2 level as well as up to threefold induction in iNOS expression compared to mock-Trx. Our results suggest that microRNAs can function as signaling molecules and identify COX-2 as an essential element in a pathway that contributes to the spread of CNS damage.

Evidently the distinguished pattern of let-7 five members’ expression in each group is directly correlated to the genetic remodeling activity that is exerted by LPS, Cur, or VPA. It is clearly demonstrated that LPS-induced neurotoxicity suppresses let-7 family miRNAs expression, an effect that is ameliorated by co-administration of either cur or VPA. Co-administration of either Cur or VPA a particular differential expression was observed in all investigated members of let-7 miRNAs confirming their function as important players in neuro-protection. Moreover a definite significant restoration of let7-a, b, and c levels were observed in VPA-Cur treated rats versus self-recovery group that initiated self-healing for 4 weeks. Treatment of induced rats with VPA or Cur alone did not induce let-7 a, b, and c in the same pattern as their combination did which indicate the synergistic effect VPA-Cur treatment. The anti-inflammatory effect of Cur is most likely mediated through its ability to inhibit COX-2 and iNOS COX-2 as important enzymes that mediate inflammatory processes. Improper upregulation of COX-2 and/or iNOS has been associated with the pathophysiology inflammatory disorders. Since Cur was shown to modulate COX-2 by direct binding, adding VPA provide synergistic effect for COX-2 and iNOS down-regulation through its activity as epigenetic modulator.

The discovery and development of miRNA-based therapeutics, as well as the diverse range of molecular cascades they can regulate, offer a new approach for treating diseases with a heterogeneic or epigenetic origin. We provide strong evidence for meaningful changes in five let-7 members miRNA expression during induction, progression, and treatment with Cur and VPA as the most salient feature. Recently, alteration of miR- let-7 members expression has also been reported in several other neurodegenerative diseases including schizophrenia, AD and addiction (Beveridge et al., 2010; Hollander et al., 2010; Santarelli et al., 2011; Wang et al., 2011). Our work thus clearly indicates that miRNAs such as let-7 members deserve further functional exploration to deepen our understanding of molecular mechanisms driving not only neuroinflammation but also other neurodegenerative disorders. It is not unlikely that future studies might reveal part of those common molecular pathways that are relevant to these conditions.

The fine-tuning activity of miRNAs has been proven crucial in the regulation of differentiation of microglia allowing the maintenance of brain homeostasis. Since a single miRNA has the capacity to target more than one protein involved in the same signaling pathway, their modulation can significantly change cell phenotypes that depend on the levels and activation of specific proteins. Such capacity reflects a molecular paradigm suitable for therapeutic intervention.

**FIGURE 8:**
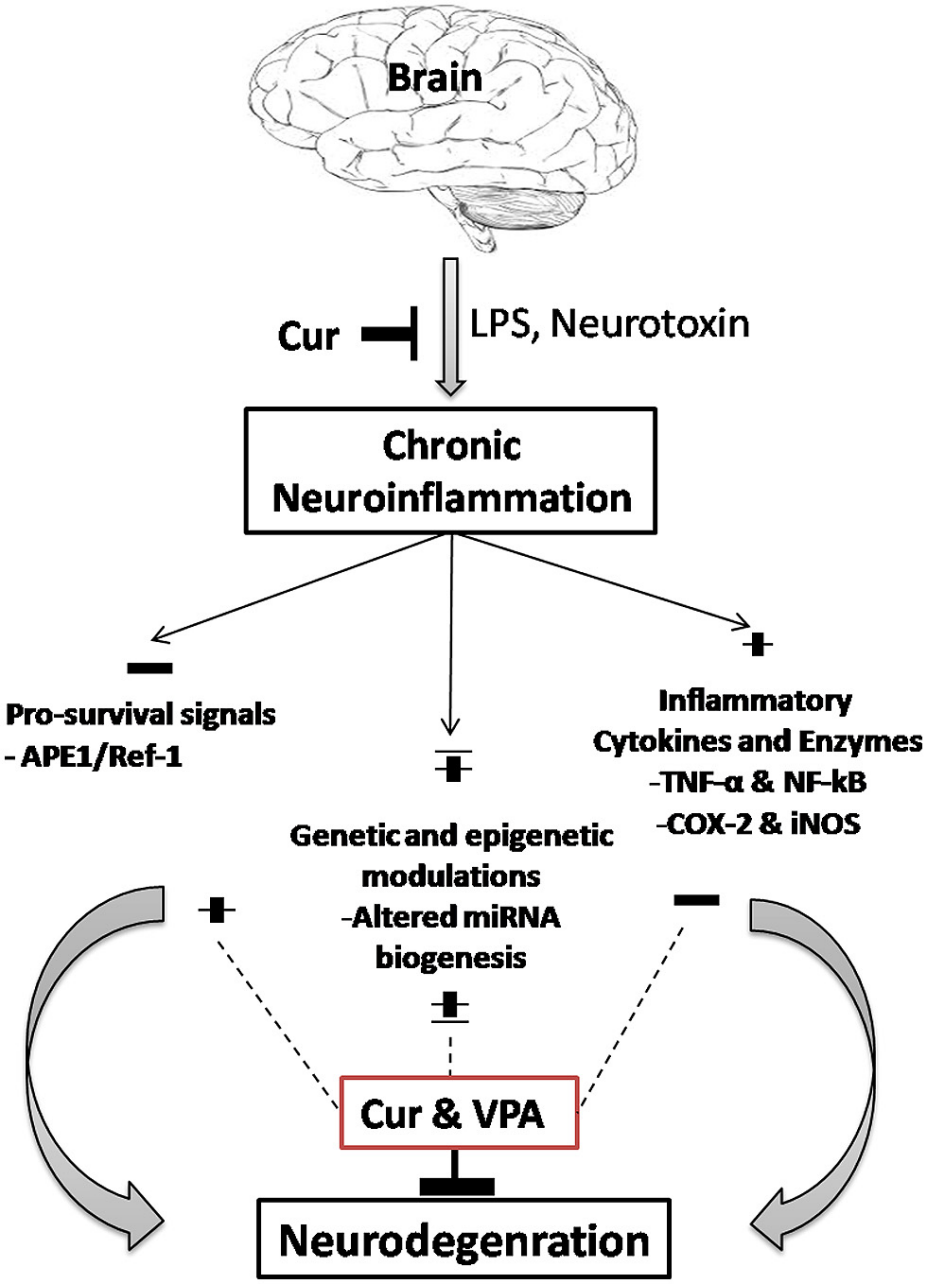
**Possible mechanisms of action of combined curcumin and VPA**.

In conclusion, as shown in **Figure 8**, the antioxidant and the current study provides evidence for the potential neuro-protective and therapeutic effect of Cur through its anti-inflammatory and gene-remodeling activities. Furthermore we highlight the synergistic effect of VPA addition to Cur for treating neuroinflammation. Also we shed the light on the role of five let-7 family in VPA-Cur mediated mechanism of actions as novel therapeutic targets. Given their important role in the regulation of gene expression, we believe that miRNA-based therapies could constitute an interesting and attractive strategy to improve microglia activity, modulating signaling pathways linked with neuroinflammation.

## Conflict of Interest Statement

The authors declare that the research was conducted in the absence of any commercial or financial relationships that could be construed as a potential conflict of interest.
